# The Management of Lamellar Macular Holes: A Review

**DOI:** 10.1155/2020/3526316

**Published:** 2020-02-21

**Authors:** Ciprian Danielescu, Horia Tudor Stanca, Florian Balta

**Affiliations:** ^1^Department of Ophthalmology, “Gr. T. Popa” University of Medicine and Pharmacy, Iasi 700111, Romania; ^2^Department of Ophthalmology, “Carol Davila” University of Medicine and Pharmacy, Bucharest 020021, Romania

## Abstract

This literature review aims to provide the retina specialist with answers to patient's questions related to the management of lamellar macular holes (LMHs). Most LMHs are stable over time, but 13–21% present an anatomic decline after 18–24 months of follow-up. Nineteen point five percent of the eyes may experience a visual acuity (VA) loss of more than 5 letters after 3 years. Many surgeons choose to perform surgery when there is significant metamorphopsia or documented decline in VA over time. The typical surgery is phacovitrectomy with the epiretinal membrane and the internal limiting membrane peeling in previously phakic eyes (41.9 to 85.3% of the eyes). In the eyes that remained phakic, cataract surgery was often necessary within the first year of follow-up (19.2 to 40% of eyes). After surgery, a VA gain was recorded in 63–94% of eyes, but some eyes (between 0 and 20%) suffered some VA loss. Progression to full-thickness macular hole may occur after surgery, and thus a second surgical intervention may be needed.

## 1. Introduction

In 1975, Gass has published a case of partial-thickness defect of the fovea (at that time considered a complication of a ruptured cyst in cystoid macular edema, CME), and named it lamellar macular hole (LMH) [1]. Later, Allen and Gass studied the mechanism of contraction of epiretinal membranes (ERMs) and concluded that a lamellar hole is the result of an abortive process in macular hole formation [2].

With the advent of optical coherence tomography (OCT), LMHs were found to present a hyporeflective area extending laterally into the foveal layers, while the external retina was spared [3]. Takahashi and Kishi described a lenticular-shaped split that was present in the inner neurosensory retina corresponding to the fovea and also documented the progression of LMH to full-thickness macular hole (FTMH) [4].

The current diagnostic criteria for LMH were defined by Witkin et al. [5] and adopted by the International Vitreomacular Traction Study Group [6]: a defect in the inner fovea with irregular foveal contour, presence of intraretinal splitting (between inner and outer foveal layers), and mostly intact photoreceptor layer.

The pathogeny of LMH is still a subject of debate. Gass has emitted the supposition that it would be caused by the spontaneous dehiscence of the wall of an intraretinal cyst, during the process of posterior vitreous detachment. The presence of an operculum in front of the fovea would confirm this pathogenic theory [7]. There are several papers describing the advent of LMHs in diseases associated with CME: post cataract surgery [1], diabetic macular edema [8], retinitis pigmentosa [9], and Coats' disease [10]. However, most cases of LMH are considered idiopathic [7] and it seems the pathogenesis of LMH cannot simply be attributed to abortive anteroposterior traction [11].

The theory of a pathogenic mechanism associated with contraction of ERMs has gained a lot of support as OCT became a standard diagnostic technique—many authors have described ERMs in virtually all cases of LMH [5, 12–16]. The presence of ERM together with an attached posterior hyaloid would cause the formation of LMH rather than FTMH because of the stabilizing effect of the posterior hyaloid on retinal structures [5]. The advent of splitting between the inner and outer retinal layers (more specifically, between the outer plexiform layer and the outer nuclear layer [17]) may prevent the dehiscence of outer layers. Linear hyperreflective structures may span the hyporeflective spaces [18].

It is important to underline the difference between LMH and macular pseudoholes (MPHs), which have a steep contour of the fovea with near-normal central foveal thickness (with no retinal tissue loss), heaped foveal edges (increased perifoveal thickness), and ERM with central opening [17, 19, 20].

For consistency, throughout this article, we have used the diagnostic criteria for LMH proposed by Witkin et al. [5, 6], even though Gaudric et al. have argued that MPH with signs of centripetal contraction of the ERM and cleavage of the foveal pit edge remain pseudoholes [21]. The case series that included both LMH and MPH were excluded unless the results of the two groups were clearly and separately presented.

Even if LMH is not a very rare occurrence, there are far fewer papers published about it compared to the body of literature on FTMHs. The evolution of untreated LMH, treatment criteria, surgical technique, and outcomes are still a matter of debate. The vitreoretinal surgeon needs that information to be systematically made available in order to recommend a course of action to the patient.

This literature review aims to provide the surgeon with a comprehensive collection of current knowledge on LMH. On one hand, we have focused on the course of untreated LMH since this information would likely be requested by the patient facing a treatment decision. On the other hand, we have systematically gathered the results of all the published surgical series (of over 10 cases), hoping to gain as much information as possible about visual outcomes and possible complications.

## 2. Methods

A PubMed search was performed using the keyword “lamellar macular holes,” and articles published in English, French, and German were included. Of the 286 published papers, we have focused on the one hand on longitudinal studies on the evolution of untreated LMH. On the other hand, we have studied the papers presenting the postoperative results (case series of at least 10 eyes). Articles presenting a cohort of untreated eyes and a cohort of operated eyes whose results were published separately were also included in this review.

### 2.1. Natural History (Observational Studies)

The prevalence of LMH in the general population ranges from 1.1% to 3.6% [7].

The first case report showed a progression of LMH to FTMH [4]. However, spontaneous closure of LMH may also be (rarely) observed [22].

The observational case series found in the literature are presented in Table 1. With one exception, the follow-up periods range between 18 and 40 months. The longest follow-up (111 months) was in a small series of 17 patients [27].

Most LMHs are morphologically stable over years of follow-up: 13% to 21% of cases show an enlargement of maximum LMH diameter and/or reduction in foveal thickness after 18–24 months [15, 24]. After the longest published follow-up (8.3 years), 1/3 of cases presented morphological progression [27].

Mean best-corrected visual acuity (BCVA) tended to be stable over time [24, 26, 28] or slowly decreased by 1 logMAR line after 8.3 years [27].

The percentage of eyes experiencing VA deterioration over 3 years was 27% (19.5% have lost 5–15 letters) [23].

García-Fernández et al. have published a cohort of patients with LMH or MPH who did not receive vitrectomy. In 30 eyes with LMH that underwent cataract surgery, the mean VA had improved from 0.51 to 0.26 logMAR. Thus, they suggested the need for phacoemulsification prior to vitreoretinal surgery in these patients [30]. Other authors have also advocated phacoemulsification before considering cataract surgery, if cataracts were suspected to be the main cause of visual loss [31].

As most observational studies were retrospective, they may be more prone to selection bias (a tendency to include mostly nonprogressive cases, and the progressive cases being operated).

### 2.2. Classification of LMHs

Romano et al. have proposed a classification of LMHs:

- Type 1: omega-shaped LMH would be caused by the evolution of a foveal pseudocyst.
- Type 2: associated with the epiretinal membrane and characterized by active tangential, multifocal contraction. The presence of vitreopapillary adhesion would increase the risk of progression.
- Type 3: without the epiretinal membrane [32].

In our experience, the vast majority of LMH would correspond to the type 2 (the only type presenting progressive changes according to Romano). This classification has not been largely adopted, and most authors preferring to differentiate LMHs by the type of preretinal tissue, as we are showing in the following.

### 2.3. Lamellar Hole-Associated Epiretinal Proliferation

In 2006, Witkin et al. wrote about some LMHs associated with ERM of “unusual thick appearance” on ultrahigh resolution-OCT [5]. In 2011, Parolini et al. differentiated ERMs into “tractional” and “dense” and found on OCT a hyporeflective material that fills the space between the dense ERM and the retinal nerve fiber layer [16].

In 2013, Bottoni categorized ERMs in “normal” and “thicker,” the latter being moderately reflective on OCT [24].

In 2014, Pang et al. described in 30.5% eyes with LMH and 8% with FTMH an entity that they named lamellar hole-associated epiretinal proliferation (LHEP): a material situated on the epiretinal surface, contiguous with the inner retinal layers, exhibiting homogenous medium reflectivity on OCT. This material appeared not to induce tractional effects [33].

In a later study (2015), the same authors found that the presence of LHEP was associated with larger LMH diameters, thinner retinal thickness at the base of the LMH, higher rates of ellipsoid zone (inner-outer segment line, IS/OS) disruption, and significantly poorer VA when compared with the eyes with LMH and without LHEP [15]. A loss of 0.3 logMAR visual acuity was found in 5% of eyes with LHEP versus 4% of eyes without LHEP over a mean retrospective follow-up of 26 months. After the acronym was coined, many authors adopted it to describe this appearance associated with LMH.

Also in 2015, Schumann et al. found that “atypical epiretinal tissue” (present in 29% of LMH eyes) was significantly associated with more IS/OS line and external limiting membrane (ELM) defects and poorer VA [34].

In 2016, Govetto et al. suggested a classification of LMH into “tractional,”characterized by schitic separation in the neurosensory retina, and “degenerative,” characterised by intraretinal cavitation in all retinal layers and often associated with nontractional epiretinal proliferation [35].

### 2.4. Surgical Technique

All the authors have performed a standard (20G to 27G) three-port pars plana vitrectomy (in phakic eyes, most authors have chosen to perform also a phacoemulsification with intraocular lens implantation). If a posterior vitreous detachment (PVD) had not already been present, it was induced by suction in front of the optic disc (many authors have used triamcinolone acetonide in order to assist the PVD). The ERMs were stained with trypan blue [25, 36, 37] or brilliant blue [38] and peeled with a forceps. The internal limiting membrane (ILM) was stained with diluted indocyanine green [39–41] or brilliant blue [36, 38], and then it was peeled.

A variability in the technique was induced by the surgeon's preferences for intravitreal tamponade: air [38, 39], isoexpansile mixture of 20% SF6 [25, 39, 40], or 14% C3F8 [36, 40–43]. The duration of face-down positioning in the presence of tamponade was 3 to 7 days (one author has instructed the patients to maintain face-down positioning for 14 days) [43]. Some authors simply advised the patient against supine positioning [36]. Michalewska et al. did not use any endotamponade [37]. Other authors have compared groups of patients operated with and without endotamponade [38, 41].

During surgery, LHEP may feel soft, “sticky,” and not easily detachable from the margins of the LMH. A suggested technique would be to start the peeling by first engaging the ILM not occupied by ERM. To reduce the risk of FTMH formation, care must be taken not to forcefully pull the ERM from the edge of the hole (Lai et al. advocated trimming of epiretinal tissue around the hole margin with scissors) [42]. Morescalchi advocated what appears to be a similar approach, that is, leaving the ILM intact for 2 disc diameters around the fovea (“foveal sparing”) [44].

Shiraga et al. suggested that the epiretinal tissue to be centripetally peeled and only to be left in the periphery of the LMH. Then, the ILM is stained and removed [45].

Recently, Takahashi has proposed the embedding of LHEP into the retinal cleavage of the LMH [46].

### 2.5. Surgical Outcomes (Interventional Studies)

The first papers describing the surgical treatment of LMH have been case reports or small case series [5, 47, 48], but after 2008, the authors have begun to publish larger case series.

We have found 19 papers reporting surgical case series of more than 10 eyes (Table 2), totalizing 729 eyes. With two exceptions, all were retrospective. The reported mean follow-up ranged from 9 to 85 months.

From a morphologic point of view, the normal foveal contour was restored in 50 to 79% of eyes.

Most authors performed phacovitrectomies in previously phakic eyes (41.9 to 85.3% of eyes) or cataract surgery during the first year of follow-up (19.2 to 40% of eyes). However, in a small case series, one author performed vitrectomy without phacoemulsification and reported that 72% of eyes gained 2 lines of VA [43].

All authors reported postoperative gains in mean VA (range 0.11 to 0.37 logMAR). The percentage of eyes with VA gain ranged from 63 to 93%. Seven articles reported the percentage of eyes that gained 2 lines of VA: between 30.4 and 93%.

In 6 papers, a VA loss after surgery was recorded in 3.8 to 20% of the eyes. Seven authors reported postoperative FTMH in 1.9 to 16.6% of the eyes. With one exception, FTMH was closed after a second procedure.

There were three reported cases of postoperative retinal detachment, resolved after a second surgery [31, 52, 53].

In the largest series (106 eyes), the causes of VA loss (20% of the eyes) were persistent foveal splitting, cystoid macular edema, papillomaculary bundle defect, retinal detachment, and FTMH, but also cataract (not operated) and in cases with long follow-up, age-related macular degeneration (AMD) [31]. The 18 eyes pseudophakic at the time of vitrectomy did not show improvement in VA (however, the 3 eyes that developed AMD and one that developed retinal detachment have probably influenced this statistic).

### 2.6. Prognostic Factors

Several papers published after 2012 dealt with the IS/OS defects, considering them associated with lower VA. Michalewska et al. suggested that eyes with IS/OS damage would have a final VA of less than 0.2 Snellen [37]. Lee et al. found that VA does not increase postoperatively in the presence of IS/OS disruption, initial VA less than 0.2 or initial foveal thickness less than 100 *μ*m [50]. They suggested that prompt surgery might be more beneficial than late intervention. Sun et al. have achieved a reduction in IS/OS defects from 63.3% preoperatively to 43% postoperatively and found a strong association between better final VA and intact IS/OS [41].

Further attention was given to the prognostic role of LHEP. Lai et al. compared a group of eyes with LMH and LHEP (where a reduction of IS/OS defects from 68.4% to 36.8% was achieved) with a group of eyes with LMH and without LHEP (where IS/OS defects diminished from 37.5% to 33.3%). The VA gain was similar in the two groups [42].

Figueroa et al. have compared eyes with “tractional” LMH (with ERMs) and “degenerative” LMH (with LHEP) finding significant improvement in VA after surgery in both groups (but higher in tractional LMH) [52].

dell'Omo pointed out that the presence of LHEP without any trace of standard ERM is rare (13.1%) and that morphological and functional outcomes after surgery did not differ in cases with and without LHEP [54].

However, Ko et al. did not obtain a significant VA improvement after surgery in eyes with LHEP [51]. Similarly, Coassin et al. reported VA improvement in eyes with “tractional” or mixed LMH, but not with “degenerative” LMH [31].

Sun et al. compared eyes operated with and without C3F8 tamponade and found no significant difference in the VA gain [41]. Similar results were reported by Sato et al. when comparing the surgical outcomes of vitrectomy with and without air tamponade [38].

## 3. Discussions

Regarding the management of a patient with LMH, the ophthalmologist must first consider that this condition may be stable for long periods of time. The proportion of eyes showing anatomical progression was 13–21% after 18–24 months of follow-up, going up to 33% after 8.3 years.

Perhaps more importantly, most studies have reported stable mean BCVA over time. Only one study has reported the proportion of eyes with VA loss: 19.5% lost 5–15 letters over the 3-year follow-up.

However, since most observational studies were retrospective, there is a possibility of selection bias (a tendency to include mostly nonprogressive cases, and the progressive cases being operated).

Taking this information into account, we have found that most authors recommend surgery when the patient complains of important metamorphopsia or there is a documented decrease in VA over time [16, 27, 36, 38, 52]. Other authors intervened with surgery whenever VA < 20/40 and there was evidence of ERM [41, 42].

It would be safe to tell our patients that all authors have reported VA benefits after surgery, with the percentage of eyes with VA gain ranging from 63% to 92%. Also, many authors have not reported cases of VA loss. However, 6 of the published papers reported small proportions (3.8 to 20%) of eyes with significant VA loss. Also, 7 surgeons encountered several cases of FTMH developed after the initial surgery, all but one closed after a second procedure. Thus, the patient must be warned that rarely a second surgery might be necessary.

LMH surgery tends to be combined with phacoemulsification in a large percentage of eyes (41.9 to 85.3%). In eyes that remain phakic after vitrectomy, most will undergo phacoemulsification in the first postoperative year (19.2 to 40% of initial patients). Therefore, one can assume that some of the VA gains are related to the phacoemulsification rather than to actual LMH surgery. Though this strategy (combining vitrectomy with phacoemulsification) appears to be efficient in our patients, with high success rates, it raises some questions regarding the actual benefit of the LMH surgery by itself.

In patients with LMH and cataract, we can at least say that the LMH does not preclude a significant improvement of VA after phacoemulsification (without vitrectomy) [30].

## 4. Conclusions

After reviewing the current knowledge about the indications and outcomes of LMH surgery, we believe that the surgeon should first inform the patient about the prognosis of the untreated condition. We found that many surgeons recommend surgery when the patient complains of significant metamorphopsia or there is evidence of VA loss over time. If LMH appears not to be the main cause of VA loss, cataract surgery may be the first (perhaps only) therapeutic step.

When surgery is performed, many surgeons would choose to combine phacoemulsification with vitrectomy and ERM and ILM peeling. Preoperative IS/OS defects are statistically associated with worse visual prognosis. The presence of LHEP is a controversial prognostic factor. Several surgical techniques have been advocated for the management of LHEP, but those techniques have not been compared to the “classic” ERM and ILM peeling.

## Figures and Tables

**Table 1:** Observational studies.

Author	No. of eyes	Mean follow-up in months (range)	Visual acuity evolution (logMAR)	Anatomical evolution	Comments
Theodossiadis et al. [23]	41	37.1 (25–54)	73% of eyes: stable VA 27% of eyes: VA loss (19.5% eyes lost 5–15 letters)	Diameter increased by 13.7%^*∗*^ Foveal thickness decreased by 10.3%^*∗*^	Metamorphopsia 75.6% eyes: initial 95% eyes: final No cataract progression

Bottoni et al. [24]	34 10 eyes with “thicker ERM”	18 (6–24)	Mean VA stable VA slightly worse at baseline^*∗∗*^	Diameter stable in 79% eyes Decrease of foveal thickness: 3% at 24 months^*∗∗*^ Thinner foveas at baseline ^*∗*^ 30% IS/OS defects (vs 12.5% in “typical ERM”)	1/10 developed FTMH (compared with 1/24 in eyes with “typical ERM”)

Celik et al. [25]	21	21.6	Initial VA 0.51 Final VA 0.55	3 had IS/OS defects at baseline ⟶ 2 developed defects	

Pang et al. [15]	145 42.7% with LHEP 57.3% ERM without LHEP	26	Initial VA 0.51 5% of eyes lost 0.3 lines Initial VA 0.33 4% of eyes lost 0.3 lines^*∗∗*^	18% anatomical progression 88% IS/OS disruption 13% anatomical progression 24% IS/OS disruption	No significant difference in the percentage of eyes that lost 0.3 logMAR lines

Zampedri et al. [26]	189 eyes Intact IS/OS line 66.1% Intact ELM 78.3% 38% “atypical ERM”	68 eyes—12 months 35 eyes—24 months	VA has not changed significantly VA slightly worse in “atypical ERM”	Mean diameter increased significantly in both groups Foveal thickness decreased significantly in the “atypical ERM” group	

Purtskhvanidze et al. [27]	17	111 (75–155)	Initial VA 0.2 Final VA 0.3	Decrease of foveal thickness^*∗*^ Increased diameter^*∗*^ Increased IS/OS defects^*∗*^	2/3 of LMH and MPH remain stable after 8.3 years

Marques et al. [28]	49 53% LHEP	32		No differences in anatomical progression in patients with or without LHEP	

Compera et al. [29]	34 (100% with LHEP)	40.5		IS/OS defects: 65% at baseline 85% at final visit	Loss of VA correlates with maximal LMH diameter and IS/OS defects

^*∗*^Statistically significant difference; ^*∗∗*^no significant difference.

**Table 2:** Interventional studies.

Author	No. of eyes (type of tamponade)	Mean follow-up in months (range)	Lens status	Mean visual acuity evolution (logMAR)	Percentage of eyes that gained /lost VA	Anatomical evolution	Comments
Garretson et al. [39]	27 (22 gas or air)	9 (2–33)		Mean improvement 3.2 lines	93% gained VA 7% lost VA	4.7% of eyes developed FTMH 92% improved OCT	

Androudi et al. [36] Prospective study	20 (C3F8)	(12–46)	60% were pseudophakic 40% phaco in the first year	Mean improvement 2.6 lines	85% gained VA	70% almost normal foveal contour 25% improved 5% no change	

Michalewska et al. [37]	26 (No tamponade)	12	19.2% phaco in the first year	Initial VA 0.2 Snellen Final VA 0.51	92% gained 2 lines 3.8% lost 2 lines	50% normal foveal contour 27% irregular foveal contour IS/OS defects: 30% initial 7.6% final	Eyes with fotoreceptor damage ⟶ final VA <0.2 Snellen

Figueroa et al. [43]	12 (C3F8)	16.1	No phaco	Initial VA 0.34 Final VA 0.17	75% gained 2 lines 25% stable VA (2 needed reoperation for FTMH) 0% lost VA	16.6% of eyes developed FTMH	

Casparis and Bovey [49]	45 (43 air or gas)		38% phaco	Initial VA 0.4 Final VA 0.13	58% gained 2 lines 0% lost VA		

Parolini et al. [16]	19 (air) (i) 13 dense ERM (ii) 6 tractional ERM	12	36% pseudophakic 64% phaco-vit	Dense ERM: Initial VA 0.4, Final VA 0.2 Tractional ERM: Initial VA 0.4, Final VA 0.2	73% gained VA 0% lost VA	15.7% of eyes developed FTMH	

Lee et al. [40]	31 (SF6)	39 (12–80)	29% were pseudophakic 41.9% phaco-vit 19.4% phaco after vitrectomy	Initial VA 0.41 3 months 0.27, 6 months 0.24, 12 months 0.22, 39 months 0.23 Mean gain 0.17	58.1% gained 2 lines 6, 5% lost VA (CMO, recurrence of LMH)	62.5% normal foveal contour 25% improved foveal appearance	

Lee et al. [50]	30		16.6% were pseudophakic 60% phaco-vit 23.3% remained phakic Cataract surgery did not correlate with final VA	Initial VA 0.51 Final VA 0.4	63% gained VA 20% stable VA 17% lost VA		In the group with intact IS/OS, VA increased from 20/50 to 20/32 VA did not increase: (i) In the group with IS/OS disruption (ii) If initial VA <0.2 Snellen (iii) If initial foveal thickness <100 μm

Celik et al. [25]	19 (SF6 or C2F6)	17.5	42% phaco	Initial VA 0.54 Final VA 0.33		10.5% of eyes developed FTMH (i) 1 reoperated successfully 5 eyes had IS/OS defects⟶ 2 were partially restored 52% normalised foveal contour 31% improvement 10% no change	Eyes with IS/OS defects did not improve VA even if defects were closed

Sun et al. [41]	30 (22 with C3F8 and 8 no tamponade)	16.9	46.6% were pseudophakic 30% phaco after vitrectomy 23.3% remained phakic	With C3F8: Initial VA 0.77 Final VA 0.44 Without C3F8: Initial VA 0.89 Final VA 0.52 Mean gain 3.4 lines	83% gained VA 63% gained 3 lines 0% lost VA	IS/OS defects: 63.3% preoperatively 43% postoperatively 73.3% restored foveal contour 16.6% improved contour 10% persistent defect 3.3% of eyes have developed FTMH	Final BCVA is associated with intact IS/OS line No significant difference in the initial and final VA between eyes with/without gas tamponade

Sato et al. [38]	41 (23 air and 18 no tamponade)	6	85.3% phaco	With air: Initial VA 0.26 Final VA 0.12 No tamponade: Initial VA 0.35 Final VA 0.14	2 lines VA gain: With air 30.4% Without air 61.1% ^*∗∗*^	IS/OS disruption in 5 eyes⟶restored postoperatively ELM disruption in 2 eyes⟶restored postoperatively	No significant difference in the initial and final VA between eyes with/without air tamponade

Lai et al. [42]	43 (C3F8) 44% LHEP 56% no LHEP	Minimum 12		Initial VA 0.78 Final VA 0.44 Initial VA 0.71 Final VA 0.42		IS/OS defects Initial 68.4 Final 36.8 Initial 37.5 Final 33.3	VA increased similarly in both groups (with/without LHEP)

Ko et al. [51]	58/73 LHEP 15/73 no LHEP	21.5	Phaco 75.9% Phaco 53.3%	Initial VA 0.3 Final VA 0.1^*∗*^ Initial VA 0.38 Final VA 0.33 ^*∗∗*^		No patient with IS/OS disruption had restored IS/OS line 4.5% developed IS/OS disruption postoperatively	Final VA significantly better in eyes without LHEP^*∗*^

Coassin et al. [31]	106 (air/SF6/C3F8)	36 (1–116)	37% phaco-vit 28% phaco after vit	Initial VA 0.45 Final VA 0.31	53% gained 2 lines 11% VA stable 20% lost VA	2.8% of eyes have developed FTMH 1 retinal detachment Restored foveal contour 66%	VA improved in tractional and mixed, but not in degenerative LMH VA did not increase in the eyes that were previously pseudophakic (18 eyes) or those that remained phakic at the end of follow-up (19 eyes)

Purtskhvanidze et al. [27]	11	85 (60–140)	28% were pseudophakic 72% phaco-vit (although they did not have cataract)	Initial VA 0.4 Immediately before vitrectomy 0.5 Final VA 0.3 ^*∗∗*^			

Figueroa et al. [52]	77 tractional LMH (with premacular membranes) 26 degenerative LMH (with LHEP)	30.8 (6–96)	12.6% phaco-vit (83.5% pseudophakic at the end)	Initial VA 0.39 Final VA 0.18 ^*∗*^ Initial VA 0.56 Final VA 0.39 ^*∗*^		14.3% initial outer retina disruption 7.7% final outer retina disruption 50% initial outer retina disruption 42.3% final outer retina disruption 1.9% of eyes have developed FTMH 1 macula-on retinal detachment	The type of tamponade did not influence anatomical success VA improvement was greater in tractional LMH

Guber et al. [53]	36	3	63.9% phaco-vit	Initial VA 0.3 Final VA 0.2	72% gained VA 19.5% stable VA 11.5% lost 1 line	92% improved foveal contour 0 IS/OS defects 1 macula-on retinal detachment	

Morescalchi et al. [44] prospective	24 degenerative LMH (with LHEP)	6		Initial VA 0.44 Final VA 0.17^*∗*^		79% restoration of foveal appearance	Fovea sparing technique (ILM left intact 2 optic disc diameters around fovea)

Takahashi et al. [46]	34 degenerative LMH (of which 10 had high myopia)	30 (12–82)	67.6% phaco-vit	Initial VA 0.31 Final VA 0.1	47% gained 2 lines 53% stable VA 0% lost 2 lines	59% recovery of ERM 47% recovery of IS/OS line	Embedding of LHEP into the retinal cleavage of the LMH

^*∗*^Statistically significant difference; ^*∗∗*^no significant difference.
